# A Comparison of Two Structurally Related Human Milk Oligosaccharide Conjugates in a Model of Diet-Induced Obesity

**DOI:** 10.3389/fimmu.2021.668217

**Published:** 2021-05-20

**Authors:** Jessica Ramadhin, Vanessa Silva-Moraes, Tamas Nagy, Thomas Norberg, Donald Harn

**Affiliations:** ^1^ Department of Infectious Diseases, University of Georgia, Athens, GA, United States; ^2^ Department of Pathology, University of Georgia, Athens, GA, United States; ^3^ Department of Chemistry-BMC, Uppsala University, Uppsala, Sweden

**Keywords:** glycoconjugates, human milk oligosaccharides (HMOs), lacto-N-fucopentaose III (LNFPIII), lacto-N-neotetraose (LNnT), Lewis^x^ antigen, metabolic syndrome, obesity

## Abstract

Obesity is the largest risk factor for the development of chronic diseases in industrialized countries. Excessive fat accumulation triggers a state of chronic low-grade inflammation to the detriment of numerous organs. To address this problem, our lab has been examining the anti-inflammatory mechanisms of two human milk oligosaccharides (HMOs), lacto-N-fucopentaose III (LNFPIII) and lacto-N-neotetraose (LNnT). LNFPIII and LNnT are HMOs that differ in structure *via* presence/absence of an α1,3-linked fucose. We utilize LNFPIII and LNnT in conjugate form, where 10-12 molecules of LNFPIII or LNnT are conjugated to a 40 kDa dextran carrier (P3DEX/NTDEX). Previous studies from our lab have shown that LNFPIII conjugates are anti-inflammatory, act on multiple cell types, and are therapeutic in a wide range of murine inflammatory disease models. The α1,3-linked fucose residue on LNFPIII makes it difficult and more expensive to synthesize. Therefore, we asked if LNnT conjugates induced similar therapeutic effects to LNFPIII. Herein, we compare the therapeutic effects of P3DEX and NTDEX in a model of diet-induced obesity (DIO). Male *C57BL/6* mice were placed on a high-fat diet for six weeks and then injected twice per week for eight weeks with 25µg of 40 kDa dextran (DEX; vehicle control), P3DEX, or NTDEX. We found that treatment with P3DEX, but not NTDEX, led to reductions in body weight, adipose tissue (AT) weights, and fasting blood glucose levels. Mice treated with P3DEX also demonstrated improvements in glucose homeostasis and insulin tolerance. Treatment with P3DEX or NTDEX also induced different profiles of serum chemokines, cytokines, adipokines, and incretin hormones, with P3DEX notably reducing circulating levels of leptin and resistin. P3DEX also reduced WAT inflammation and hepatic lipid accumulation, whereas NTDEX seemed to worsen these parameters. These results suggest that the small structural difference between P3DEX and NTDEX has significant effects on the conjugates’ therapeutic abilities. Future work will focus on identifying the receptors for these conjugates and delineating the mechanisms by which P3DEX and NTDEX exert their effects.

## Introduction

Obesity and related metabolic syndrome (MS) pose major medical risks to those afflicted, often worsening outcomes to infectious diseases (i.e., COVID-19) or leading to type 2 diabetes mellitus (T2DM), cardiovascular disease, fatty liver disease, stroke, and cancer. As of 2017-2018, the age-adjusted prevalence of obesity was estimated to be 42.4%. This is an 11.9% increase from 1999-2000, suggesting that the epidemic of being obese is worsening as time progresses (1). Obesity is defined as having a body-mass index (BMI) ≥ 30 kg/m^2^, but this measure is insufficient when used as a sole indicator for classification (2). MS is defined as having at least three of the following criteria: waist circumference >102cm for males; >88cm for women, elevated blood glucose levels >100mg/dL, decreased HDL cholesterol <50mg/dL for males; <40mg/dL for females, elevated triglycerides >150mg/dL, or elevated blood pressure >130/85 (3). The International Diabetes Foundation (IDF) has also suggested inclusion of additional criteria, such as elevated circulating levels of CRP, TNFα, IL-6 (4, 5). Once an individual is diagnosed with MS, his/her risk of serious disease is heightened.

Metabolic inflammation is a sustained, low-grade immune response that occurs as consequence of excess nutrient consumption and has been identified as the nexus between the obese state and serious complications. Within adipose tissue (AT) depots, adipocytes (fat cells) expand in number (hyperplasia) and size (hypertrophy) to store lipids and prevent lipotoxic build-up in peripheral organs (i.e. liver, pancreas, skeletal muscle, etc.) (6–11). This protective effect subsides, however, once adipocytes encounter mechanical stress and hypoxic conditions as a result of overexpansion (12–14). This leads to an increase in detrimental adipokines, chemokines, and cytokines, a decrease in anti-inflammatory or insulin-sensitizing factors, immune cell infiltration, and insulin resistance (IR) (15–20). In this regard, targeting altered signaling or cellular composition of obese AT might have therapeutic potential for those with MS.

We have been examining the mechanisms and biological effects of two human milk oligosaccharides (HMOs), lacto-N-fucopentaose III (LNFPIII) and lacto-N-neotetraose (LNnT). HMOs are the third most abundant component of human milk and provide numerous protective benefits to the breastfeeding infant (i.e. providing nutrients, training the immune system, preventing infection, establishing the microbiome, etc.) (21, 22). LNFPIII and LNnT differ in structure *via* the presence/absence of an α1,3-linked fucose residue. Due to the α1,3-linked fucose residue, LNFPIII is difficult to produce *via* chemical or enzymatic methods. LNnT has been synthesized by various laboratories and is present at higher concentrations (0.74 g/L vs. 0.33 g/L) in human milk (23–26). Glycom A/S has registered LNnT for use in infant formula in Europe (Novel Food Application 157) and the United States (GRAS Notice 659) (6). Pre-clinical assessment has been conducted on a chemically synthesized version of LNnT and there were no adverse effects at doses of up to 5000mg/kg/day in rats (26). Oral supplementation with LNnT has also been shown to be well-tolerated in humans (27).

LNnT is easier to synthesize and has been shown to be well-tolerated in humans, therefore we asked if the small structural difference between LNFPIII and LNnT impacts the conjugates’ therapeutic effect in DIO mice. LNFPIII conjugates (P3DEX) and LNnT conjugates (NTDEX) are composed of 10-12 molecules of LNFPIII or LNnT attached to a 40 kDa dextran carrier *via* an acetylphenylenediamine (APD) linker. Previous studies have shown that therapeutic intervention with LNFPIII-Dex (25µg/dose) in diet-induced obese (DIO) mice twice per week for four weeks led to improved metabolic homeostasis and increased concentrations of circulating IL-10. Of note, P3DEX treatment improved glucose and insulin tolerance, as well as enhanced insulin signaling in WAT. This was shown *via* increased expression of insulin receptor β (*insrb*), insulin receptor substrate 2 (*irs2*), CCAAT/enhancer-binding protein α (*cebpa*), and glucose transporter 4 (*glut4*). P3DEX treatment also decreased macrophage infiltration and crown-like structures in WAT. This coincided with decreased expression of tumor-necrosis factor α (*tnfa*), caspase-1 (*casp1*), NLR family pyrin domain containing 3 (nlrp3), interleukin-18 (il18), and interleukin-1β (*il1b*). In addition to restoring metabolic homeostasis and ameliorating insulin resistance, LNFPIII-Dex treatment also decreased lipogenic genes (*fas*, *acc1/2*, *scd1*, and *srebp1c*) and fat accumulation in the liver (28).

We report here that treatment with P3DEX, but not NTDEX, decreases total weight gain, reduces AT, improves glucose tolerance, and ameliorates insulin resistance. P3DEX and NTDEX exert wide-ranging effects on circulating chemokines, cytokines, adipokines, and incretin hormones when compared to DIO control mice treated with the 40 kDa dextran (DEX) carrier. Most striking, P3DEX, but not NTDEX, reduces WAT inflammation and hepatic lipid accumulation. This suggests that the slight structural difference between P3DEX and NTDEX alters the conjugates therapeutic abilities and exemplifies the differential roles that individual HMOs might execute *in vivo.*

## Materials and Methods

### Preparation of HMO Conjugates

LNFPIII was synthesized by Dr. Peng George Wang (Georgia State University, Atlanta, GA) (23, 29). LNnT was synthesized by Neose Technologies, Inc. LNFPIII (MW: 853.877 g/mol) and LNnT (MW: 707.60 g/mol) were sent to Dr. Thomas Norberg (Uppsala University, Uppsala, Sweden) for conjugation to aminodextran (DEX, 40 kDa average Mw, from Invitrogen, prod # D1861) using APD linker-spacers. On average, conjugates had 10-12 LNFPIII or LNnT monomers per 40 kDa dextran carrier. LNFPIII accounts for ~17-20% of the molecular weight of the P3DEX conjugate. LNnT accounts for ~15-17% of the molecular weight of the NTDEX conjugate.

### Animal Experiments

**Figure 1:**
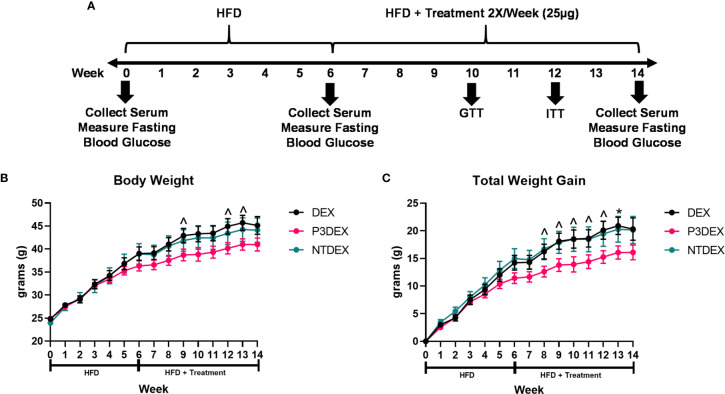
LNFPIII conjugates reduce total body and organ weights. **(A)** Experimental design. Male *C57BL/6* mice were placed on HFD for 6 weeks prior to intervention twice per week with 25μg DEX, P3DEX, or NTDEX. **(B, C)** P3DEX reduces total body weight and weight gain. Two-way ANOVA with Dunnett’s Multiple Comparisons Test. **(D–F)** P3DEX reduces scWAT and WAT, but not BAT. **(G–J)** P3DEX and NTDEX have no effect on liver, heart, spleen, and kidney weights. One-way ANOVA with Dunnett’s Multiple Comparisons Test. *indicates a significant difference between DEX and P3DEX (p ≤ 0.05). ^indicates a trend between DEX and P3DEX (p ≤ 0.10).

6 to 8-week old male *C57BL/6* mice were purchased from The Jackson Laboratory and maintained on a 12h light/dark cycle in the University of Georgia’s AALAC-accredited College of Veterinary Medicine Animal Resources Facility with food and water available *ad libitum*. After one week of acclimation, mice were housed n=3/cage and placed on a high-fat diet (HFD: Bio-Serv Cat. No. F3282) as described in Bhargava et al. (28). Given that the HFD is subject to spoilage, food was replenished 3 times per week. After 6 weeks of HFD, mice were divided into 3 cohorts and injected twice per week for 8 weeks *via* the intraperitoneal route with 25µg of 40 kDa dextran (DEX), LNFPIII conjugated to 40 kDa dextran (P3DEX), or LNnT conjugated to 40 kDa dextran (NTDEX). An experimental timeline is shown in **Figure 1A**. DEX, P3DEX, and NTDEX were dissolved in 0.9% NaCl prior to injection in a volume of 200µL. Experiments were performed in two independent mouse cohorts (n=6-8/group). Metabolic studies (GTT/ITT) were performed after 4 weeks of treatment with a 2-week rest period between *in vivo* assays. We performed a glucose tolerance test (GTT) at W10 of the experiment and an insulin tolerance test (ITT) at W12 (described below). Body weights were measured once/week and fasting blood glucose levels were measured prior to start of HFD (W0), pre-treatment (W6), and post-treatment (W14). Mice were euthanized at the end of W14 *via* CO_2_ asphyxiation followed by cervical dislocation after a 6h fast. Organs and serum samples were collected and stored at -80°C until use.

### Measurement of Body and Organ Weights

Mice were placed in a clean plastic container on a tared compact scale (Ohaus, Cat. No. CS200) to acquire body weight measurements (in grams). Body weights were recorded each week on Monday afternoon to minimize daily fluctuations. Body weights were recorded to the nearest hundredth. At sacrifice, organs (i.e. heart, spleen, liver, kidney, AT) were placed on clean Fisherbrand™ Polystyrene Antistatic Weighing Dishes (Fisher Scientific, Cat. No. 08-732-112) on a tared analytical scale (Sartorius, Cat. No. TE313S) for measurement. Organ weights were recorded to the nearest thousandth.

### Glucose Tolerance Test (GTT)

A glucose tolerance test (GTT) were performed after 4 weeks of treatment (W10), according to Beguinot & Nigro with some modifications (24). Mice were fasted for 6h to obtain baseline blood measurements. Various fasting periods have been utilized in published studies involving GTTs and ITTs, but 6h fasts appear to be more physiological than longer 14-16h fasts. Previous studies have shown that prolonged fasting in mice results in increased insulin sensitivity, whereas fasting in humans leads to inhibition of insulin-stimulated glucose uptake (25, 26). Moreover, prolonged fasting in mice may lead to a state of starvation (30). To ensure translatable results, we utilized a 6h fasting period in our experiments. During GTTs, blood was acquired *via* tail snip and measured using a Bayer Contour^®^ Next blood glucose monitoring system (Bayer, Parsippany, NJ). Basal glucose measurements were taken before mice were administered 2g glucose/kg (Sigma, Cat. No. G8270) in 10µL/g body weight *via* oral gavage. Thereafter, blood glucose measurements were taken at 15, 30, 60, 90, 120, 150, and 180-minute timepoints.

### Insulin Tolerance Test (ITT)

An insulin tolerance test (ITT) was performed after 6 weeks of treatment (W12) to allow for a 2-week rest period between metabolic tests. The ITT was also performed according to Beguinot & Nigro with the aforementioned fasting modification (24). During the ITT, mice were fasted for 6h prior to baseline blood glucose measurements and intraperitoneal injection of 0.5IU/kg insulin (Humulin^®^ R U-100, Lilly, Cat. No. HI-210) diluted in 0.9% NaCl in an injection volume of 3.6µL/g body weight. Thereafter, blood glucose measurements were taken at 15, 30, 60, and 90-minute timepoints. If blood glucose levels dropped below 36mg/dL, mice were rescued *via* injection of 20% aqueous glucose solution (Sigma, Cat. No. G8270).

### Serum Evaluation of Adipokines, Chemokines, and Cytokines

The Bio-Plex Pro Mouse Cytokine 23-Plex Assay (Bio-Rad, Cat. No. M60009RDPD) and the Bio-Plex Pro Mouse Diabetes 8-Plex Assay (Bio-Rad, Cat. No. 171F7001M) were performed in multi-plex, according to Bio-Rad’s Technical Note 5975. These two panels can be performed *via* multiplex without altering assay sensitivity, specificity, and accuracy. Serum samples collected at sacrifice after a 6h fast and frozen at -80°C were prepared according to manufacturer’s instructions. Adipokines, chemokines, and cytokines were detected and quantified using the Bio-Rad Bio-Plex^®^ 200 System, available at UGA’s Cytometry Shared Resource Laboratory.

### Histopathology

WAT and liver samples were preserved in 10% formalin and processed, sectioned, and stained with hematoxylin & eosin (H&E) by the Comparative Pathology Lab in the College of Veterinary Medicine at the University of Georgia. Dr. Tamas Nagy, a board-certified pathologist, then assessed inflammation with WAT sections and lipid accumulation in the liver.

### Measurement of ALT and AST

Liver function was assessed *via* measurement of alanine aminotransferase (AST) and aspartate aminotransferase (ALT). ALT was measured in serum using the Alanine Aminotransferase (ALT or SGPT) Activity Colorimeteric/Fluorometric Assay Kit (Biovision, Cat. No. K752), according to manufacturer’s instructions. AST was measured in serum using the Aspartate Aminotransferase (AST or SGOT) Activity Colorimetric Assay Kit (Biovision, Cat. No K753), according to manufacturer’s instructions. Absorbance was measured using the SPECTROstar Nano Microplate Reader (BMG Labtech).

### Statistical Analysis

Statistics were performed using One-Way or Two-Way ANOVA with Dunnett’s Multiple Comparisons Tests in GraphPad Prism 8. * or ** was used to indicate significance (*p<0.05 or **p<0.01). ^ was used to indicate a trend (^p<0.10). Values are presented as mean ± SEM. Experiments were repeated twice for a total n=12-14 per treatment group.

## Results

### LNFPIII Conjugates Reduce Total Body and Organ Weights

Male *C57BL/6* mice were placed on HFD for 6 weeks (W0-6) prior to beginning treatment twice per week with 25ug DEX, P3DEX, or NTDEX (W7-14) *via* the intraperitoneal route. Body weight was monitored weekly throughout the duration of the experiment (W0-W14). An experimental timeline is shown in **Figure 1A**. Mice treated with P3DEX weighed less overall than mice treated with the DEX carrier control or NTDEX (**Figure 1B**). Compared to DEX, P3DEX treatment reduced total body weight. This reduction trended towards significance (Two-Way ANOVA with Dunnett’s Multiple Comparisons Test; ^<0.10) at W9, 12, and 13. While it appears that the DIO mice treated with P3DEX begin to show lessened body weight and reduced total weight gain at W5 and W6 of the experiment, the difference is neither trending, nor significant at these timepoints. In **Figure 1B**, statistical analysis performed at W5 between DEX and P3DEX resulted in a non-trending or non-significant p-value of 0.5482 (One-Way ANOVA with Dunnett’s Multiple Comparisons Test, *p<0.05, ^p<0.10). At W6, the p-value for the statistical test performed between DEX and P3DEX was equal to 0.2719 (One-Way ANOVA with Dunnett’s Multiple Comparisons Test, ^p<0.10, *p<0.05). Treatment with NTDEX did not cause a significant reduction in total body weight at any point of measurement. Furthermore, there was no significant difference in total body weight between the 3 groups prior to treatment. When normalizing the body weights and examining total weight gain over time, it is clear that P3DEX treatment led to a reduction in total weight gain when compared to DEX-treated animals (**Figure 1B**). This trended toward significance at W8, 9, 10, 11, and 12 (Two-Way ANOVA with Dunnett’s Multiple Comparisons Test; *<0.05, ^p<0.10), and became significant at W13 (Two-Way ANOVA with Dunnett’s Multiple Comparisons Test; *p<0.05, ^p<0.10). When we normalized the body weight in **Figure 1C**, the difference between DEX and P3DEX remained non-trending and non-significant at W5 and W6, with a p-value of 0.4307 and 0.1891, respectively (One-Way ANOVA with Dunnett’s Multiple Comparisons Test, *p<0.05, p<0.10). For these reasons, we are confident that the trending and significant differences we observe at later timepoints are divergent and a result of P3DEX intervention. Again, treatment with NTDEX did not cause a significant reduction in total weight gain at any point of measurement and nearly mirrors the DEX control.

At sacrifice (W14), we collected and weighed subcutaneous (scWAT), visceral (VAT), and brown (BAT) adipose tissue, as well as livers, hearts, spleens, and kidneys. **Figure 1D** demonstrates that we saw a trend towards reduction of scWAT in P3DEX-treated mice compared to those treated with DEX (One-Way ANOVA with Dunnett’s Multiple Comparisons Test; *p<0.05, ^p<0.10). We saw no effect of NTDEX on scWAT compared to DEX. In terms of vWAT, we noted a significant reduction in P3DEX-treated mice compared to those treated with DEX (**Figure 1E**). We observed no differences in BAT between groups, but this is a smaller AT depot (**Figure 1F**). We did not observe a significant difference in liver, heart, spleen, or kidney weights (**Figures 1G–J**).

### LNFPIII Conjugates Improve Glucose Tolerance and Reduce Insulin Resistance

**Figure 2:**
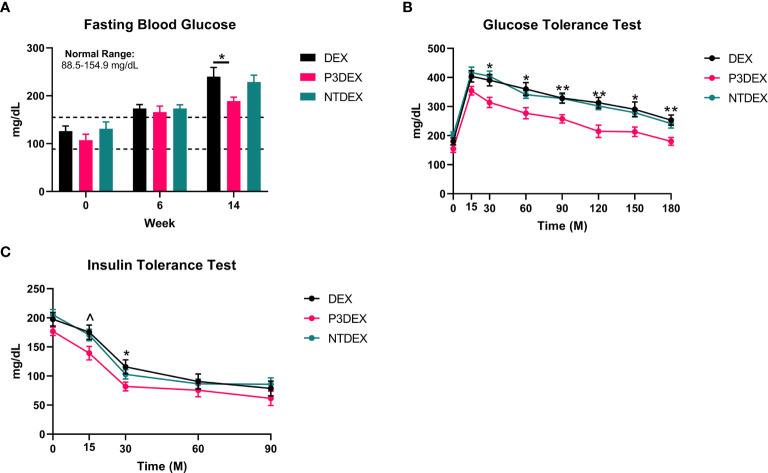
LNFPIII conjugates improve glucose homeostasis and reduce insulin resistance. **(A)** P3DEX reduces fasting blood glucose levels. **(B)** P3DEX improves glucose tolerance. **(C)** P3DEX improves insulin sensitivity. Two-way ANOVA with Dunnett’s Multiple Comparisons Test. *indicates a significant difference between DEX and P3DEX (p ≤ 0.05). ^indicates a trend between DEX and P3DEX (p ≤ 0.10). (**p ≤ 0.01).

We measured 6h-fasting blood glucose levels prior to HFD (W0), prior to treatment (W6), and prior to sacrifice (W14). Prior to HFD (W0), the average fasting blood glucose for all male mice was within normal range (88.5-154.9 mg/dL) (31). This was elevated prior to treatment (W6), demonstrating that the HFD induced metabolic dysfunction. Prior to sacrifice and post-treatment, mice treated with P3DEX had significantly lower fasting blood glucose levels (**Figure 2A**) than those treated with DEX. NTDEX treatment did not result in reductions in fasting blood glucose levels compared to those treated with DEX. When administered an oral gavage of 2g glucose/kg body weight during a GTT, mice treated with P3DEX exhibited a less dramatic increase in blood glucose levels at the 15-minute timepoint compared to those treated with DEX or NTDEX. P3DEX-treated animals were able to return to basal blood glucose levels more quickly than DEX- or NTDEX-treated mice. Mice treated with P3DEX had significantly lower blood glucose levels throughout the duration of the GTT and this was significant at 30, 60, 90, 120, 150, and 180 minutes (**Figure 2B**). When given an intraperitoneal injection of 0.5IU/kg body weight insulin, mice treated with P3DEX exhibited a greater decrease in blood glucose levels overall compared to those treated with DEX or NTDEX. Mice treated with P3DEX had significantly lower blood glucose levels throughout the duration of the ITT and this trended towards significance at 15 minutes (**Figure 2C**). Blood glucose levels of P3DEX-treated mice was significantly lower than DEX-treated mice at the 30-minute timepoint (**Figure 2C**). Thereafter, blood glucose levels between groups appeared to stabilize. This shows that DIO mice treated with DEX or NTDEX were less responsive than mice treated with P3DEX when injected with a bolus of insulin, suggesting the presence of IR in the DEX and NTDEX groups.

### LNFPIII and LNnT Conjugates Alter Hematopoietic Signals

Individuals diagnosed with MS are at higher risk of infection and illness, suggesting that the constant presence of low-grade inflammation exhausts and dampens the immune response to other pathogens or insults (32, 33). In order to maintain function, progenitor cells depend on nutrients and hematopoietic signals to proliferate and differentiate into diverse white blood cells (WBC) populations (34, 35). IL-3, for example, promotes proliferation of hematopoietic cells (34, 36). G-CSF and GM-CSF are then responsible for differentiation of cells into specific lineages (i.e. granulocytes and monocytes) and subsequent activation (34, 37). High levels of IL-3, G-CSF, or GM-CSF can lead to increased populations of WBCs, which can exacerbate inflammation and damage normal tissues. High WBC counts have been associated with parameters related to inflammation and well-documented in obese subjects (38–43).

**Figure 3:**
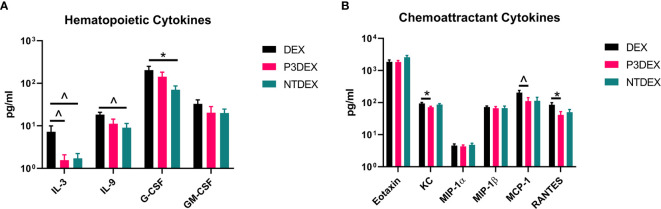
LNFPIII and LNnT conjugates alter hematopoietic and chemoattractant signals. Measurements of **(A)** hematopoietic and **(B)** chemoattractant cytokines are shown here in response to DEX, P3DEX, or NTDEX treatment. One-way ANOVA with Dunnett’s Multiple Comparisons Test. *indicates a significant difference (p ≤ 0.05). ^indicates a trend (p ≤ 0.10).

We measured IL-3, C-CSF, and GM-CSF in serum samples collected from fasting DIO mice treated with DEX, P3DEX, or NTDEX. Serum from DIO mice treated with P3DEX or NTDEX trended towards decreased IL-3 levels compared to the DEX control (**Figure 3A**). G-CSF and GM-CSF were also slightly reduced, with the reduction in G-CSF becoming significant following NTDEX treatment (**Figure 3A**). These results suggest that both P3DEX and NTDEX have potential to dampen the excessive immune response associated with the obese state.

### LNFPIII and LNnT Conjugates Alter Chemoattractant Signals

WBCs depend on chemoattractant signals for migration to injured sites. In the obese state, expansion of WAT enhances chemokine release and attracts WBCs to AT depots (44). This worsens metabolic inflammation and associated comorbidities.

We measured eotaxin/CCL11, KC/CXCL1, MCP-1/CCL2, MIP-1α/CCL3, MIP-1β/CCL4, and RANTES in serum samples collected from fasting DIO mice treated with DEX, P3DEX, or NTDEX. We saw no differences in circulating levels of eotaxin/CCL11, a chemokine known to attract eosinophils (**Figure 3B**). While circulating eotaxin/CCL11 is known to increase in the obese state, neither P3DEX, nor NTDEX altered these levels (45, 46). This makes sense as we have no evidence that either conjugate alters eosinophil function. KC/CXCL1 is a neutrophil chemoattractant that increases in patients with T2DM, as well as in diabetic-prone *db/db* mice with evidence of impact on pancreatic islet function (47–49). P3DEX treatment resulted in a significant decrease in serum KC/CXCL1 compared to the DEX control (**Figure 3B**). NTDEX treatment did not reduce serum KC/CXCL1, suggesting mechanistic differences between NTDEX and P3DEX conjugates. It is well-reported in the literature that P3DEX and NTDEX act on macrophages (50–54). MCP-1/CCL2 is instrumental for macrophage recruitment to AT depots and circulating concentrations are increased in the obese state (55). Furthermore, mice deficient in MCP-1 signaling exhibit lessened macrophage infiltration and inflammation in AT depots (56, 57). P3DEX treatment resulted in a significant decrease in serum MCP-1/CCL2 compared to the DEX control (**Figure 3B**). Although NTDEX also appeared to decrease circulating MCP-1/CCL2 levels, this decrease was not significant (**Figure 3B**). Neither P3DEX, nor NTDEX had an effect on MIP-1α/CCL3 or MIP-1β/CCL4 (**Figure 3B**). MIP-1α and MIP-1β are also elevated in genetic (*ob/ob*, *db/db*) and DIO mice, as well as obese humans (19, 58, 59). While MIP-1α and MIP-1β both increase in the obese state, there is evidence that they do not alter macrophage infiltration in AT. RANTES is responsible for T cell recruitment and is also increased in the obese state (58, 59). We noted a significant reduction in serum RANTES following P3DEX treatment (**Figure 3B**). NTDEX also appeared to reduce serum concentration of RANTES, but this was not significant (**Figure 3B**). Overall, these results demonstrate that P3DEX is more effective at decreasing circulating chemoattractant signals than NTDEX.

### LNFPIII and LNnT Alter Cytokines Involved in Innate and Adaptive Immunity

**Figure 4:**
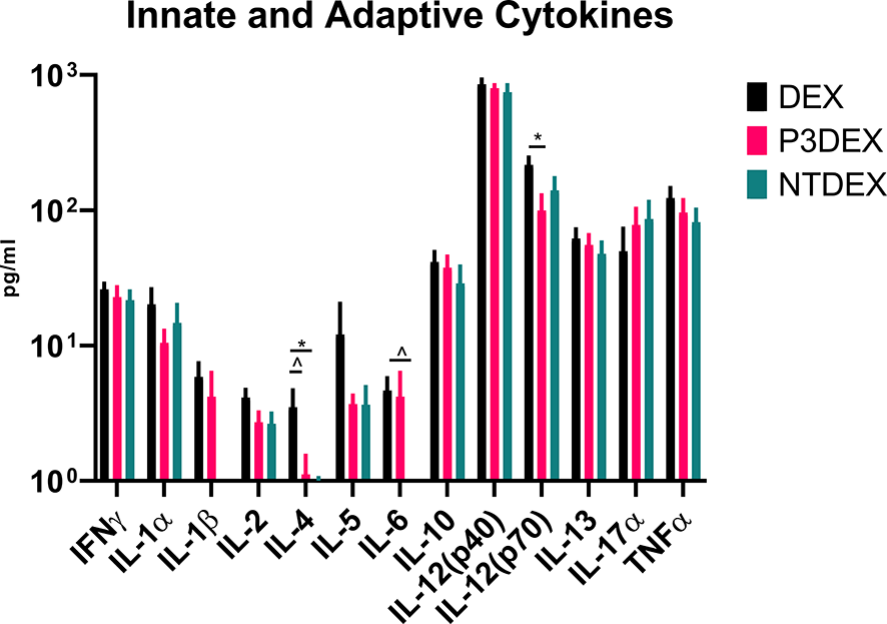
LNFPIII and LNnT conjugates alter innate and adaptive cytokines. Measurement of innate and adaptive cytokines are shown here in response to DEX, P3DEX, or NTDEX treatment. One-way ANOVA with Dunnett’s Multiple Comparisons Test. *indicates a significant difference (p ≤ 0.05). ^indicates a trend (p ≤ 0.10).

Given their pleiotropic nature, cytokines are difficult to characterize in the context of DIO. We measured numerous innate and adaptive cytokines in serum samples collected from fasting DIO mice treated with DEX, P3DEX, or NTDEX. Once macrophages are recruited to AT depots, IFNγ secretion helps these cells maintain classical (M1) activation (60). Neither P3DEX, nor NTDEX, had a significant effect on circulating concentrations of IFNγ (**Figure 4**). P3DEX treatment led to a slight, nonsignificant, reduction in IL-1α (**Figure 4**). IL-1β was unaffected by P3DEX or NTDEX treatment (**Figure 4**). Both IL-1α and IL-1β are elevated in obese individuals with reductions documented following weight loss (61–63). IL-2, involved in T cell proliferation and activation, was lowered, but nonsignificant, in response to P3DEX and NTDEX (**Figure 4**). IL-4 trended towards reduction in P3DEX-treated mice and was significant for those treated with NTDEX (**Figure 4**). This reduction was unanticipated, given that both P3DEX and NTDEX promote T_H_2 responses and alternative activation (M2) of macrophages (51–54). P3DEX and NTDEX treatment also led to slight reductions in IL-5, another T_H_2 cytokine, which tends to help maintain homeostasis in WAT depots (**Figure 4**) (64). P3DEX did not impact circulating IL-6 levels, but NTDEX eliminated IL-6 compared to the DEX control (**Figure 4**). IL-9, a T_H_2 cytokine, trended toward decreased circulating levels in DIO mice treated with NTDEX (**Figure 4**) (65). Neither P3DEX, nor NTDEX treatment, had a significant effect on circulating IL-10 levels (**Figure 4**). While P3DEX and NTDEX did not impact levels of IL-12(p40), P3DEX treatment led to a significant reduction in IL-12(p70) (**Figure 4**). This reduction in the bioactive form of IL-12 suggests quelling of the T_H_1 immune response, which might contribute to P3DEX’s therapeutic effects (66). We observed no differences in serum IL-13, another T_H_2 cytokine that aids in overcoming insulin resistance (**Figure 4**) (67). IL-17a increased in response to P3DEX and NTDEX treatment, but this increase was not significant (**Figure 4**). Last, we observed a slight, but nonsignificant, reduction in TNFα in response to P3DEX and NTDEX treatment (**Figure 4**). Similar to other markers, circulating TNFα is elevated in obese individuals (17). Moreover, neutralization of TNFα led to improved insulin responses (15). Overall, P3DEX treatment seems to decrease circulating cytokine levels, both inflammatory and TH_2_-associated. While these cytokine responses are ambiguous when considered on their own, they are indispensable for delineating how P3DEX treatment reduces body weight and improves glucose homeostasis *in vivo*.

### LNFPIII Conjugates Modulate Adipokine Secretion

**Figure 5:**
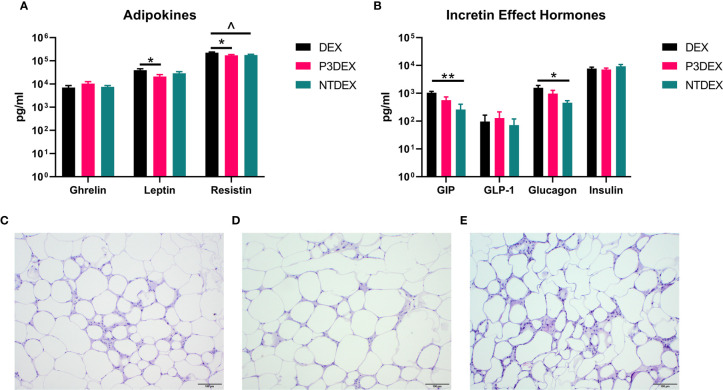
LNFPIII conjugates reduce adipokines and WAT inflammation, while LNnT conjugates alter incretin effect hormones. Measurement of **(A)** adipokines and **(B)** incretin effect hormones in DIO mice treated with DEX, P3DEX, or NTDEX. One-way ANOVA with Dunnett’s Multiple Comparisons Test. *indicates a significant difference (*p ≤ 0.05, **p ≤ 0.01). ^indicates a trend (p ≤ 0.10). Histological analysis of vWAT depots from DIO mice treated with **(C)** DEX, **(D)** P3DEX, or **(E)** NTDEX.

Measurement of AT-specific markers (adipokines), rather than pleiotropic cytokines, might provide more insight on the therapeutic or non-therapeutic effects of P3DEX and NTDEX in the context of DIO. Ghrelin, for instance, is an orexigenic adipokine that stimulates food intake (68). Circulating levels of ghrelin are lower in the obese state and higher in those that are lean (69–71). Ghrelin also quells secretion of markers related to inflammation and inhibits NF-κB signaling (72, 73). Although we were unable to monitor food intake in our studies, we found that P3DEX treatment led to a slight, but nonsignificant, increase in ghrelin (**Figure 5A**). P3DEX treatment also led to a significant decrease in leptin, a hormone generated in AT in proportion to fat content (**Figure 5A**) (74). Circulating leptin concentrations coincide with reductions in AT (75, 76). Decreases in leptin also coincide with increases in orexigenic peptides (i.e. ghrelin), which might explain the slight increase shown in **Figure 5A** (77, 78). NTDEX treatment led to a trending reduction in circulating levels of resistin, while P3DEX treatment led to a significant decrease (**Figure 5A**). P3DEX treatment seems to have beneficial effects on specific adipokines (i.e. leptin and resistin), which corresponds with its effects on adipose tissue and glucose homeostasis.

### LNnT Conjugates Alter the Incretin Effect

During the incretin effect, glucose-dependent insulinotropic polypeptide (GIP) and glucagon-like peptide 1 (GLP-1) are released from the gut to stimulate insulin secretion in response to food intake (79). P3DEX and NTDEX reduced circulating levels of GIP, with the reduction induced *via* NTDEX being significant (**Figure 5B**). In pancreatic islets, decreases in GIP correspond with decreases in insulin and glucagon secretion (80). Both P3DEX and NTDEX did not have an effect on GLP-1, another incretin hormone (**Figure 5B**). P3DEX treatment led to a slight reduction in glucagon and NTDEX treatment led to a significant reduction (**Figure 5B**). NTDEX treatment led to a significant reduction in GIP, which aligns with the corresponding decrease in glucagon. We observed a trending increase in the ratio of insulin to glucagon in response to NTDEX treatment, an indicator of excess nutrient load (**Figure 5B**) (81). We conclude here that P3DEX treatment has beneficial effects on specific adipokines (i.e. ghrelin, leptin, and resistin), while NTDEX might impact incretin hormones and postprandial insulin release. These findings further support that these two HMO conjugates differ in terms of mechanism and therapeutic potential.

### LNFPIII Conjugates Reduce WAT Inflammation and Hepatic Lipid Accumulation

**Figure 6:**
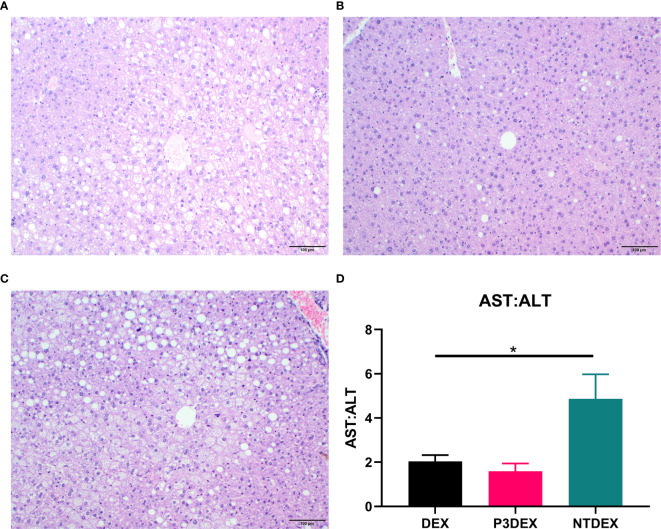
LNFPIII conjugates reduce hepatic lipid accumulation and liver damage. Liver sections from DIO mice treated with **(A)** DEX, **(B)** P3DEX, or **(C)** NTDEX. **(D)** Ratio of circulating AST:ALT. One-way ANOVA with Dunnett’s Multiple Comparisons Test. *indicates a significant difference (p ≤ 0.05).

We report various *in vivo* changes in response to both P3DEX and NTDEX. Upon examination of vWAT tissue, we observed what appeared to decreased immune cell infiltrate and crown-like structures in DIO mice treated with P3DEX compared to DEX and NTDEX (**Figures 5C–E**). DIO mice treated with NTDEX exhibited greater inflammation within vWAT even when compared to the DEX control (**Figure 5E**). In the liver, we observed significant lipid accumulation in DIO mice treated with the DEX control (**Figure 6A**). In contrast, we observed reduced lipid accumulation in the livers of DIO mice treated with P3DEX (**Figure 6B**). DIO mice treated with NTDEX showed greater lipid accumulation than the DEX control, suggesting a worsening of MS (**Figure 6C**). This was corroborated by the increased ratio of aspartate aminotransferase (AST) to alanine aminotransferase (ALT) detected in the serum of DIO mice treated with NTDEX (**Figure 6D**). At the tissue level, it is clear that NTDEX does not induce the same therapeutic benefit as P3DEX.

## Discussion

Although LNFPIII and LNnT differ only by the presence or absence of an α1,3-linked fucose in their structures, their therapeutic effects in a DIO model differ significantly *in vivo*. In general, HMOs present in human breastmilk are known to block infection, modulate the immune response, shape the intestinal microbiome, and serve as nutrients for brain development (22, 82). However, the effects of specific groups of HMOs (nonfucosylated neutral HMOs, fucosylated HMOs, and sialylated HMOs) or individual HMOs themselves have not been thoroughly studied. 2’-fucosyllactose (2’FL; fucosylated) and LNnT (neutral) are the two most abundant and well-studied HMOs to date [Ref]. 2’FL has been reported at ~2.74g/L in secretor mothers, while LNnT has been reported at ~0.74g/L (21). Both HMOs have been purified from human breastmilk and/or synthesized at industrial levels for supplementation in infant formula (23, 29, 83–85). LNFPIII, in contrast, is less abundant (~0.33g/L) and expensive to acquire from purified human breastmilk (21). It has also not been synthesized *via* chemical or enzymatic methods at an industrial level. To determine if LNnT would function *in vivo* similar to LNFPIII, we initiated studies to compare the therapeutic effects of LNFPIII conjugates to LNnT conjugates in a murine model of DIO.

Previous studies demonstrate both LNFPIII and LNnT conjugates promote M2 macrophage polarization, an immune process that has been deemed important for regulating and ameliorating adipose tissue inflammation and treating T2DM (51, 52, 86, 87). Several anti-diabetic drugs on the market, such as metformin and several thiazolidinediones target insulin resistance in part *via* altering M1/M2 macrophage polarization and reducing inflammation within WAT (88, 89). Bhargava et al. (2012) demonstrated that LNFPIII conjugates improve glucose tolerance and insulin resistance, as well as reduce WAT inflammation and ameliorate non-alcoholic hepatosteatosis in a similar model of DIO (28). We expand on these studies herein, demonstrating that DIO mice treated with LNFPIII conjugates (P3DEX) exhibit reductions in total weight gain and subcutaneous/visceral AT (**Figures 1B–E**). Reductions in total weight gain and subcutaneous/visceral AT depots were not reported in Bhargava et al. (2012), but it is important to note the difference in treatment duration between the two studies. In Bhargava et al. (2012), DIO mice treated with LNFPIII conjugates twice per week for four weeks. The experimental timeline here differs, during which treatment was performed twice per week for eight weeks. It is possible that the longer duration of treatment used in this experiment allowed for differences in weight gain and subcutaneous/visceral AT depots to become apparent. Similar to Bhargava et al. (2012), we observed significant reductions in fasting blood glucose levels post-treatment (**Figure 2A**), as well as improved glucose and insulin tolerance (**Figures 2B, C**). It is striking that the LNnT conjugates (NTDEX) did not induce these effects. We have shown in previous studies that P3DEX acts on B cells, macrophages, dendritic cells, adipocytes, and hepatocytes (28, 50, 52–54, 86, 90–94). NTDEX activates macrophages, but not dendritic cells, suggesting differences in cellular mechanisms between the two glycans (51). We also have evidence that P3DEX and NTDEX act on adipocytes *in vitro* (unpublished). Thus, it is likely that M2 macrophage polarization is not the sole mechanism *via* which P3DEX exerts its therapeutic effect. Furthermore, additional studies must be performed in parallel to delineate the mechanistic differences present between the two HMO conjugates.

This is the first *in vivo* report comparing the therapeutic effects of P3DEX and NTDEX in a DIO model. Compared to P3DEX, NTDEX did not reduce weight gain or improve glucose homeostasis. Both P3DEX and NTDEX had differential effects on circulating chemokines, cytokines, adipokines, and incretin hormones (**Figures 3** **–** **5**). Only P3DEX reduced weight gain and improved glucose homeostasis, yet both P3DEX and NTDEX altered cytokines involved in hematopoiesis and may each have roles in reducing inflammation (**Figures 1** **–** **3**). The effects of P3DEX appear to be more pronounced as treatment of DIO mice led to reductions in several chemotactic cytokines (i.e. KC, MCP-1, RANTES) (**Figure 3**). This could be due to reductions in AT mass and subsequent decreases in pro-inflammatory signals from AT depots. P3DEX and NTDEX also altered numerous innate and adaptive cytokines, but given the pleiotropic nature of these markers, it is difficult to make conclusions about the role that they might have in ameliorating MS (**Figure 4**). Furthermore, an increase/decrease in one marker can lead to a compensating increase/decrease in another. We saw a trending decrease in IL-4 for animals treated with P3DEX and a significant decrease for those treated with NTDEX. P3DEX and NTDEX both promote T_H_2 responses and alternative activation (M2) of macrophages, so this result was unexpected (51–54). The role of IL-6 in DIO is controversial, as some studies suggest that IL-6 exacerbates insulin resistance and others note beneficial effects (95–97). Given that we do not observe improvements in glucose or insulin tolerance in DIO mice treated with NTDEX, it is likely that elimination of IL-6 does not have a significant therapeutic impact in our studies. Furthermore, deletion of IL-6 in *in vivo* studies have not been successful in delineating its role in DIO and T2DM (97). P3DEX has been shown to increase circulating IL-10 levels in DIO mice, but the studies presented herein are different in terms of experimental conditions and design (i.e. animal housing facilities, longer treatment period, etc.) (28). IL-17a is a prominent marker of inflammation and is elevated in obese individuals, but there is also evidence of an anti-adipogenic role for this cytokine (98). The fasting serum samples analyzed here offer a snapshot in time following 8 weeks of DEX, P3DEX, or NTDEX treatment. It is well-known that these markers fluctuate over time and/or depend on disease progression. Another method to determine the specific roles and importance of the documented adipokines, chemokines, or cytokines would be to eliminate these molecules in DIO mice on DEX, P3DEX, and NTDEX treatment regimens and evaluate whether the conjugates’ therapeutic capabilities are altered.

While the chemokine and cytokine results do not allow us to focus on a distinct mechanism, it is clear that P3DEX and NTDEX altered adipokines and incretin hormones in a more straight-forward manner (**Figure 5**). Adipokines, such as ghrelin, leptin, and resistin, are directly secreted from AT depots and indispensable for metabolic regulation. We observed a slight, but non-significant increase in ghrelin in response to P3DEX treatment (**Figure 5A**). Ghrelin is known to decrease in the obese state and inversely correlates with BMI, so the observed increase in response to P3DEX treatment aligns with the decreased weight gain that we report here (99–101). In the obese state, individuals often present with low ghrelin levels and high leptin levels to which they become resistant (102). P3DEX slightly increased circulating ghrelin, as well as significantly decreased circulating leptin, an adipokine generated in proportion to fat content (**Figure 5A**) (103). P3DEX also significantly decreased resistin, an adipokine known to increase with inflammation and insulin resistance (**Figure 5A**) (104). Resistin also perpetuates states of inflammation *via* induction of pro-inflammatory cytokines (i.e. IL-1, IL-6, IL-12, TNFα, etc.) and molecules related to chemoattraction (i.e. VCAM-1, ICAM-1, MCP-1, etc.) (105, 106). Whether these changes occur as a result of overall decreases in AT or if P3DEX acts directly on adipocytes to modulate ghrelin, leptin, and resistin secretion is yet to be investigated. In contrast to P3DEX, NTDEX induced significant changes in incretin hormones. NTDEX treatment led to a significant decrease in GIP, a hormone that stimulates insulin secretion and synthesis, as well as glucagon secretion (**Figure 5B**) (80). In this regard, the observed decrease in response to NTDEX treatment could be damaging in the context of DIO. This decrease also corresponded with a significant decrease in glucagon secretion (**Figure 5B**) and a trending increase in the ratio of insulin:glucagon (**Figure 5B**).

Similar to Bhargava et al. (2012), P3DEX decreased WAT inflammation and lipid accumulation in the liver (**Figures 5**, **6**). However, NTDEX treatment seemed to worsen WAT inflammation and increase hepatic lipid accumulation even compared to DEX treatment (**Figures 5**, **6**). This was exemplified by the significant increase in the ratio of AST : ALT in DIO mice treated with NTDEX (**Figure 6D**). We expected NTDEX to ameliorate WAT inflammation and hepatic lipid accumulation similar to P3DEX, however, NTDEX appeared to have a damaging effect at the tissue level. Future studies will further investigate mechanistic changes induced by P3DEX and NTDEX at the tissue level in this DIO model.

The complete mechanism of both LNFPIII and LNnT conjugates is unknown. Internalization of LNFPIII conjugates occurs *via* a receptor-mediated process, which undergoes clathrin-dependent endocytosis (53). Internalization does not require TLR4 or MyD88, but does require CD14. Signaling continues *via* CD14/TLR4-Ras-Raf1-TPL2-MEK to induce ERK and NFκB signaling. This results in production of anti-inflammatory mediators, such as IL-4, IL-10, MMP9, and CCL22. In contrast, LNnT conjugates do not activate antigen-presenting cells (APCs) *via* TLR4/MD2/CD14, so it is probable that the fucose residue on LNFPII is vital for signaling (54). It is also likely that, while anti-inflammatory *in vivo*, LNnT conjugates induce effects that differ from LNFPIII conjugates. LNFPIII conjugates also act directly on adipocytes and hepatocytes, but it is unknown whether this is the case for LNnT conjugates. To further investigate the mechanism of LNFPIII and LNnT conjugates, we have generated monoclonal antibodies (mAbs) that will serve as useful probes for future studies (i.e. co-immunoprecipitation). We hope to determine the receptors that P3DEX and NTDEX bind to in various cells, elucidate their overall mechanisms, and determine if P3DEX might be a useful treatment for humans with MS.

## Data Availability Statement

The raw data supporting the conclusions of this article will be made available by the authors, without undue reservation.

## Ethics Statement

The animal study was reviewed and approved by UGA’s Institutional Animal Care and Use Committee (IACUC).

## Author Contributions

JR and DH designed the experiments. JR and VS-M performed the experiments. TNa performed histopathological analysis. JR and DH analyzed and interpreted the results. JR and DH wrote the manuscript. TNo synthesized both LNFPIII and LNnT conjugates. All authors contributed to the article and approved the submitted version.

## Funding

This project was funded by an American Heart Association (AHA) Predoctoral Fellowship (17PRE33410423) and UGA’s Obesity Initiative.

## Conflict of Interest

The authors declare that the research was conducted in the absence of any commercial or financial relationships that could be construed as a potential conflict of interest.
